# R-CEOP as first-line treatment for anthracycline-ineligible patients with diffuse large B-cell lymphoma

**DOI:** 10.1038/s41408-022-00723-4

**Published:** 2022-09-02

**Authors:** Diana Al-Sarayfi, Frederik O. Meeuwes, Müjde Durmaz, Djamila E. Issa, Rolf E. Brouwer, Aart Beeker, Anna van Rhenen, Pim G. N. J. Mutsaers, Lara H. Böhmer, Marjolein W. M. van der Poel, Liane te Boome, Tom van Meerten, Martine E. D. Chamuleau, Josée M. Zijlstra, Mirian Brink, Marcel Nijland

**Affiliations:** 1 Department of Hematology, University Medical Center Groningen, Groningen, The Netherlands; 2 Department of Hematology, Treant Hospital, Emmen, The Netherlands; 3 Department of Research and Development, Netherlands Comprehensive Cancer Organisation (IKNL), Utrecht, The Netherlands; 4 Department of Internal Medicine, Jeroen Bosch Hospital, ‘s-Hertogenbosch, Netherlands; 5 Department of Internal Medicine, Reinier de Graaf Gasthuis, Delft, The Netherlands; 6 Department of Oncology, Spaarne Gasthuis, Hoofddorp, The Netherlands; 7 Department of Hematology, Cancer Center, UMC Utrecht, Utrecht, The Netherlands; 8 Department of Hematology, Erasmus MC Cancer Institute, Rotterdam, The Netherlands; 9 Department of Hematology, Haga Teaching Hospital, The Hague, The Netherlands; 10 Department of Internal Medicine, Division of Hematology, GROW School for Oncology and Developmental Biology, Maastricht University Medical Center, Maastricht, The Netherlands; 11 Department of Hematology, Haaglanden Medical Center, The Hague, The Netherlands; 12 Department of Hematology, Cancer Center Amsterdam, Amsterdam University Medical Center, Vrije Universiteit Amsterdam, Amsterdam, The Netherlands

**Keywords:** B-cell lymphoma, Epidemiology

Dear Editor,

Diffuse large B-cell lymphoma (DLBCL) is the most frequently diagnosed non-Hodgkin lymphoma (NHL) [1]. Rituximab, cyclophosphamide, doxorubicin, vincristine and prednisone (R-CHOP) is the standard treatment for DLBCL. Nevertheless, doxorubicin, like all anthracyclines, is associated with dose-dependent cardiotoxicity [2]. Particularly in patients with congestive heart failure and patients previously exposed to anthracyclines, the use of doxorubicin is contraindicated. In the Netherlands, doxorubicin is most frequently replaced by etoposide (R-CEOP) for anthracyclines-ineligible patients, but efficacy data regarding this regimen are scarce. Recently, two population-based studies from Canada reported on the outcome of R-CEOP. R-CEOP was feasible, although the two studies showed conflicting results [3, 4]. While one study showed inferior outcome of patients treated with R-CEOP, the other observed no difference in disease-specific survival (DSS) between R-CEOP and R-CHOP. Randomized clinical trials (RCT) would be needed to evaluate the efficacy of R-CEOP unbiased, compared to R-CHOP. However, RCTs among anthracyclines-ineligible patients would be unethical, as patients randomized to the doxorubicin-group would experience severe cardiotoxicity due to their cardiac dysfunction. Therefore, propensity-score-matching using population-based data is needed. The aim of this population-based study was to determine the efficacy of R-CEOP in anthracycline-ineligible patients with DLBCL.

We identified all patients ≥18 years diagnosed with DLBCL who received at least one cycle of R-CHOP or R-CEOP between 2014 and 2018, using the Netherlands Cancer Registry (NCR) [5]. Information on patient characteristics and treatment is routinely recorded in the NCR by trained registrars of the NCR through retrospective medical records review. Since 2014, the Lugano classification has been used by the physicians for response evaluation in the Netherlands. Information on the last known vital status for all patients (i.e. alive, death, or emigration) is obtained through annual linkage with the Nationwide Population Registries Network that holds vital statistics on all residents in the Netherlands. Patients alive were censored on February 1st, 2021.

The primary endpoints were progression-free survival (PFS), overall survival (OS) and relative survival (RS). OS was defined as the time between diagnosis and death from any cause, and PFS as the time between diagnosis and tumor progression or death, whichever occurred first. RS was defined as the ratio of the overall survival (OS) of the patient cohort to the expected OS of an equivalent group from the general population, matched to the patients by age, sex, and calendar year. As such, RS reflects the overall excess mortality associated with a DLBCL diagnosis, thereby estimating DSS in the absence of information on the cause of death. The secondary endpoint was overall response rate (ORR), defined as a response of partial remission of the disease or better.

Patients were assigned to the R-CEOP group when they had received 50% or more of their cycles with etoposide instead of doxorubicin. Patients with R-CEOP were propensity-score-matched with patients with R-CHOP in a 1:4 ratio, including age, sex, Ann Arbor stage and International Prognostic Index (IPI) score to account for baseline differences. The log-rank test was used to evaluate differences in survival distributions. Uni-and multivariable proportional hazards regression analyses were performed to assess the impact of treatment on survival after adjustment of sex, age, Ann Arbor stage, IPI score and number of cycles, thereby calculating hazard ratios (HR) and corresponding 95% confidence intervals (95% CI). The Privacy Review Board of the NCR approved the use of anonymous data for this study.

Between 2014 and 2018, 87 DLBCL patients with R-CEOP were matched to 333 DLBCL patients treated with R-CHOP and were included in our study. Median age of the total group of patients was 74 years (range, 39–91 years) and 67% had an advanced stage (Ann Arbor ≥ 3). A total of 42 (48%) patients had high-risk disease (IPI score ≥ 3). Of the 87 patients treated with R-CEOP, 27 patients (31%) had a prior malignancy, as compared to 73 patients (22%) treated with R-CHOP. Baseline characteristics according to R-CEOP and R-CHOP are presented in Table 1.

**Table 1:** Characteristics of patients with a diffuse large B-cell lymphoma receiving R-CEOP or R-CHOP.

Characteristics				
	R-CEOP		R-CHOP	
	n	(%)	n	(%)
**Total no. of patients (row %)**	87	(21)	333	(79)
**Sex**				
Female	34	(39)	127	(38)
Male	53	(61)	206	(62)
**Age, years**				
Median (range)	74 (39–91)		73 (22–95)	
18–60	16	(18)	59	(18)
≥61	71	(82)	274	(82)
**WHO performance status**				
0–2	56	(64)	194	(58)
3–4	4	(5)	8	(2)
Unknown	27	(31)	131	(39)
**Ann Arbor stage**				
I, II	28	(32)	105	(32)
III, IV	58	(67)	228	(68)
Unknown	1	(1)	0	(0)
**Elevated LDH**				
No	43	(49)	144	(43)
Yes	43	(49)	182	(55)
Unknown	1	(1)	7	(2)
**>1 extranodal localizations**				
No	65	(75)	231	(69)
Yes	22	(25)	102	(31)
**IPI score**				
0–2	45	(52)	168	(50)
3–5	42	(48)	165	(50)
**Number of cycles**				
≥6 cycles	61	(70)	231	(69)
<6 cycles	26	(30)	102	(31)

Abbreviations: *WHO* World Health Organization, *IPI* International Prognostic Index, and *LDH* lactate dehydrogenase.

The median number of cycles administered was 6 (range, 1–8) for both R-CEOP and R-CHOP regimens. Of the 87 patients treated with R-CEOP, 67 (77%) received R-CEOP only, and 20 patients (23%) received both R-CEOP and R-CHOP, of whom the median number of R-CEOP cycles was 5 (range, 3–8) and of R-CHOP 2 (range, 1–4). Among the patients treated with R-CEOP, 13 patients (15%) received subsequent radiotherapy, as compared to 48 patients (14%) in the R-CHOP group. Three patients (3%) treated with R-CEOP received CNS-prophylaxis, as compared to 21 patients (6%) treated with R-CHOP.

The ORR was not significantly different between patients treated with R-CEOP and patients treated with R-CHOP (75% vs. 83%, respectively; *p* = 0.15; Fig. 1). The CR rates in the R-CEOP and R-CHOP groups were 61% and 72%, respectively (*p* = 0.21).

**Fig. 1:**
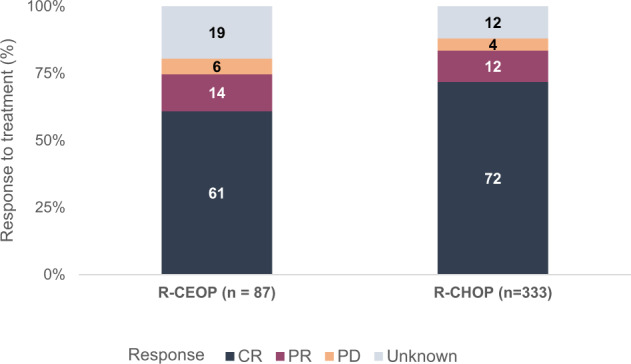
Best observed response in patients with diffuse large B-cell lymphoma treated with R-CEOP and R-CHOP. Stacked bar graph depicting best response showing no significant difference in overall response in patients treated with RCEOPas compared to patients treated with R-CHOP (*p* = 0.15).

The median follow-up was 38 months (interquartile range [IQR], 16–58 months). The 4-year PFS was inferior for patients treated with R-CEOP (44%; 95% CI, 33%–55%) as compared to R-CHOP (58%; 95% CI, 52%–63%; *p* = 0.03; Fig. 2A). The 4-year OS was 48% (95% CI, 36%–59%) in the R-CEOP group *vs*. 62% (95% CI, 57%–68%; *p* = 0.05; Fig. 2B) in the R-CHOP group. However, the 4-year RS was not statistical significantly different for patients treated with R-CEOP (54%; 95% CI, 41%–67%) as compared to patients treated with R-CHOP (71%; 95% CI, 65%–77%; *p* = 0.77; Fig. 2C). In multivariable analysis, risks of mortality (HR, 1.4; 95% CI, 1.00–2.03) and relapse (HR, 1.4; 95% CI 1.02–2.00) were increased for patients treated with R-CEOP as compared to patients treated with R-CHOP, and were negatively affected by male gender, Ann Arbor stage III/IV, IPI score ≥3 and <6 cycles of treatment (Table 2).

**Fig. 2:**
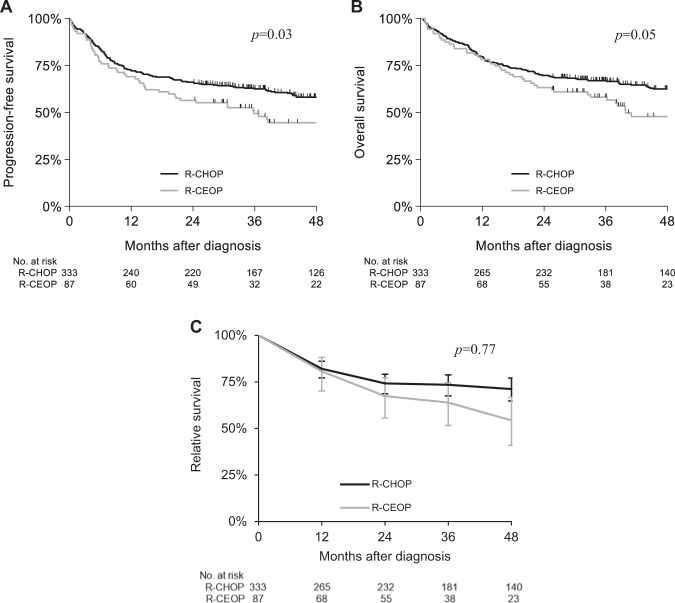
Survival analysis. **A** Progression-free survival (PFS) of patients with diffuse large B-cell lymphoma (DLBCL) treated with R-CEOP and R-CHOP. Kaplan–Meier curves showing significant inferior 4-year PFS in patients with DLBCL treated with R-CEOP (*p* = 0.03). **B** Overall survival (OS) of patients with diffuse large B-cell lymphoma (DLBCL) treated with R-CEOP and R-CHOP. Kaplan–Meier curves showing a significant inferior 4-year OS in patients with DLBCL treated with R-CEOP (*p* = 0.05). **C** Relative survival (RS) of patients with diffuse large B-cell lymphoma (DLBCL) treated with R-CEOP and R-CHOP. Kaplan–Meier curves showing no significant difference in 4-year RS in patients with DLBCL treated with R-CEOP (*p* = 0.77).

**Table 2:** Results of the uni- and multivariable Cox regression analysis on progression-free survival and overall survival for patient with diffuse large B-cell lymphoma treated with R-CEOP versus R-CHOP.

Covariate	Univariable						Multivariable					
	PFS			OS			PFS			OS		
	HR	95% CI	*P*-value^b^	HR	95% CI	*P*-value^b^	HR^a^	95% CI	*P*-value^b^	HR^a^	95% CI	*P-*value^b^
**R-CEOP**												
No	1	Reference		1	Reference		1	Reference		1	Reference	
Yes	1.43	1.02–2.00	0.04	1.43	1.00–2.03	0.05	1.55	1.11–2.17	0.01	1.58	1.11–2.25	0.01
**Sex**												
Female	1	Reference		1	Reference		1	Reference		1	Reference	
Male	1.54	1.12–2.10	<0.01	1.48	1.07–2.06	0.02	1.50	1.09–2.06	0.01	1.40	1.00–1.95	0.05
**Age**												
18–60	1	Reference		1	Reference							
≥61	1.94	1.22–3.09	<0.01	2.30	1.37–3.86	<0.01						
**Ann arbor stage**												
I, II	1	Reference		1	Reference		1	Reference		1	Reference	
III, IV	2.17	1.51–3.12	<0.01	2.13	1.45–3.12	<0.01	2.03	1.25–3.30	<0.01	1.87	1.11–3.16	0.02
**IPI score**												
0–2	1	Reference		1	Reference		1	Reference		1	Reference	
3–5	2.12	1.57–2.86	<0.01	2.28	1.65–3.14	<0.01	1.79	1.20–2.67	<0.01	2.16	1.39–3.36	0.01
**Number of cycles**												
≥6 cycles	1	Reference		1	Reference		1	Reference		1	Reference	
<6 cycles	2.36	1.76–3.17	<0.01	2.95	2.16–4.01	<0.01	3.30	2.42–4.49	<0.01	4.25	3.08–5.87	<0.01

Abbreviations: *IPI* International Prognostic Index, *CI* confidence interval.

^a^Each covariate is simultaneously adjusted for all other covariates in the table.

^b^*P*-values are compared with the reference category.

In this Dutch population-based study among patients diagnosed with DLBCL, we demonstrate that OS is inferior for patients treated with R-CEOP as compared to R-CHOP, but not for RS. Our findings are in line with previous studies that demonstrated significant differences in OS between patients receiving R-CEOP and R-CHOP [3, 4]. The observed OS difference between patients treated with R-CEOP and R-CHOP in these studies was larger (18–20%) compared to our results (14%). While Moccia et al. reported similar 5-years DSS between the treatment regimens, Puckrin et al. showed a significant survival benefit for R-CHOP over R-CEOP with 4-years DSS estimates of 69 and 48%, respectively. The similar 4-year RS estimates for both treatment groups in our study indicates that the observed difference in OS is most likely not related to lymphoma. A single-arm study of 26 patients treated with R-CEOP showed a significantly better survival outcome in patients with a germinal center B cell [6]. However, data among cell of origin (COO) was only available in 12 patients. Furthermore, Puckrin et al. did not report a difference in survival outcomes between COO.

There are several other potential treatment options for newly diagnosed DLBCL patients with a contraindication for doxorubicin. Non-pegylated liposomal doxorubicin has been assessed in patients diagnosed with DLBCL, with no difference observed in cardiotoxicity [7, 8]. In combination with rituximab, anthracycline-free regimens include cyclophosphamide, vincristine and prednisone (R-COP), gemcitabine and oxaliplatin (R-GemOx), lenalidomide (R2), bendamustine, gemcitabine, cyclophosphamide, vincristine, and prednisolone (R-GCVP) [9–12]. And while these regimens are generally well tolerated showing 2-year OS ranging from 38 to 65%, these are considered palliative options. More recently, mosunetuzumab [13], a bispecific antibody (CD3xCD20), and pixantrone [14] have shown encouraging results with tolerable toxicity in unfit and anthracyclines-ineligible DLBCL patients, respectively.

The main strength of this study was the use of a nationwide population-based cancer registry. As mentioned earlier, to correct for indication bias, propensity-score-matching was performed. MYC-status as well as history of cancer were not included as matching characteristics since MYC-status was only routinely performed following the World Health Organization (WHO) classification of 2017, and type of prior cancer was heterogenous between R-CEOP and R-CHOP suggesting major differences in outcome. Limitations of our study mainly pertain to the lack of detailed information on comorbidities e.g., congestive heart failure and cardiotoxicity. Identification of these factors may lead to improved understanding of individualized therapeutic intervention choices by the physicians, pinpointing the prior usage of anthracyclines and thereby minimizing residual confounding. Due to lacking information on cause of death in the NCR, RS, which is considered the gold-standard for performing a cause-specific survival analysis, was used to estimate DSS. With the expected disbalance in comorbidities between patients with R-CEOP and R-CHOP, but with similar RS, we assume that the OS difference is lower in clinical practice. Despite these limitations, this gives insight into the outcome of patient groups usually not eligible for clinical trials.

In conclusion, this nationwide, population-based study shows that R-CEOP is an alternative treatment for anthracycline-ineligible patients. With similar RS rates among the two regimens in our study, it is most likely that the difference in OS is due to comorbidities. R-CEOP is the treatment of choice for anthracycline-ineligible patients.

## Data Availability

The data that support the findings of this study are available via The Netherlands Comprehensive Cancer Organisation. These data are not publicly available, and restrictions apply to the availability of the data used for the current study. However, these data are available upon reasonable request and with permission of The Netherlands Comprehensive Cancer Organisation.
